# Prognostic value of different cardiac magnetic resonance imaging derived parameters in Egyptian patients with ST-elevation myocardial infarction after successful reperfusion by primary percutaneous intervention

**DOI:** 10.1186/s43044-019-0035-x

**Published:** 2019-12-21

**Authors:** Diaa Kamal, Ahmed S. Ibrahim, Merhan Ahmed Nasr, Sherihan S. Madkour

**Affiliations:** 1 Cardiology department, Faculty of medicine, Ain Shams University, Abbasia Street, Cairo, Egypt; 2 Radiodiagnosis Department, Faculty of medicine, Ain Shams University, Cairo,, Egypt

**Keywords:** ST-elevation myocardial infarction, Primary percutaneous coronary intervention, Cardiac magnetic resonance, Major adverse cardiovascular events

## Abstract

**Background:**

Cardiac magnetic resonance (CMR) is an extremely accurate and useful modality that can give much data about myocardial damage after acute myocardial infarction and consequently can give a good idea about long-term prognosis. Unfortunately, this modality is still underused in Egypt. We tried to assess the prognostic significance of different parameters derived from CMR in Egyptian patients presenting with ST-elevation myocardial infarction (STEMI) treated by primary percutaneous intervention (PPCI). Twenty-five patients who presented with acute STEMI and were successfully reperfused by PPCI within 12 h from symptoms onset were included. CMR was performed 2–4 days after PPCI. Six months of long-term follow-up for major adverse cardiovascular events (re-infarction, new-onset heart failure and cardiac death) was done. CMR-derived parameters (edema volume, area at risk, infarction volume, infarction percentage, microvascular obstruction volume, microvascular obstruction percentage, myocardial salvage and myocardial salvage index) were analyzed in relation to incidence of major adverse cardiovascular events (MACE).

**Results:**

Seven patients suffered from MACE. Univariate logistic regression analysis showed a significant correlation between edema volume (*P* = 0.04), area at risk (*P* = 0.05), infarction percentage (*P* = 0.05) and the occurrence of MACE. Multivariate logistic regression analysis showed that infarction percentage (*P* = 0.05) is the best parameter that can predict MACE.

**Conclusion:**

Infarction percentage is potentially the most important prognosticator derived from CMR in Egyptian patients with acute STEMI successfully reperfused by PPCI.

## Background

Ischemic heart disease (IHD) is the leading cause of death worldwide and also in Egypt [1, 2]. Deaths due to coronary artery disease (CAD) reached 24.58% of total deaths in Egypt according to the most recent data from the World Health Organization (WHO) [1]. In the last decade, a good decline in short- and long-term mortality after ST-elevation myocardial infarction (STEMI) is obvious owing to the extended use of primary percutaneous coronary intervention (PPCI), thrombolytic therapies, modern antithrombotics and measures of secondary prevention [3]. Management of STEMI patients during this period is also improving in Egypt with an increase in the number of patients treated with PPCI yielding results that can be compared to Western data [4].

However, this favorable decline is simultaneously generating an increasing cohort of STEMI survivors who are prone to long-term mechanical and arrhythmic hazards [5]. Identification of high-risk STEMI patients is very important to risk stratify them and properly incorporate them into secondary prevention programs to avoid complications. Up till now, all practice guidelines utilize post-MI echocardiography-derived left ventricular ejection fraction (EF) as the most important measure for risk stratification of STEMI patients [6, 7]. Echocardiography in the context of acute MI has important limitations related to myocardial stunning and compensatory hyperkinesia of healthy myocardium that can make its value as an important prognosticator debatable [8].

Cardiac magnetic resonance (CMR) is an imaging modality that is gaining an increasing interest from investigators in the field of post-STEMI risk stratification owing to its ability to accurately describe and quantify the irreversible damage that affected the infarcted myocardium and consequently predict adverse clinical outcomes beside its ability to evaluate ventricular functions and morphology [9].

Unfortunately, till now there is no consensus about which CMR-derived parameter can be used as the best prognosticator that can be used accurately to risk stratify patients after STEMI.

In our small study, we tried to get preliminary data about the prognostic significance of different CMR-derived parameters in Egyptian patients presenting with STEMI treated by PPCI.

## Methods

This study was a prospective observational study that was conducted upon 25 patients presented to the cardiology department in Ain Shams University hospitals in the time interval from August 2016 to January 2017.

The study was approved by the Research Ethics Committee (Faculty of Medicine, Ain Shams University, FWA 00006444) and all patients signed an informed consent for participation in the study in accordance with the Declaration of Helsinki. We included patients presented with acute STEMI (any territory) within the first 12 h after onset of chest pain who were candidates for PPCI according to the most recent practice guidelines [6, 7]. There was no age or sex predilection.

### Exclusion criteria

Patients with delayed presentation (> 12 h), any previous history of ischemic heart diseases, hemodynamic instability, renal impairment (serum creatinine > 2 mg/dl), contraindications to MRI (claustrophobia, an implanted metallic device, pacemakers …), pregnant and lactating females were excluded from the study.

Eligible patients presented with acute STEMI were directly transferred with close monitoring to the catheterization laboratory after doing a rapid thorough clinical examination and signing an informed consent. Rapid evaluation with bedside echocardiography to exclude mechanical complications was done for all patients. All patients received an oral loading dose of Aspirin 300 mg and Clopidogrel 600 mg. Coronary angiography was done rapidly by operators highly experienced in PPCI using adequate orthogonal views to detect the culprit lesion. PPCI was done aiming at flow restoration in the culprit artery. Patients were then transferred to coronary care unit (CCU) under close monitoring and were kept on anti-ischemic measures.

All relevant characteristics of our patients were recorded including demographic characteristics, presence of major cardiovascular risk factors, myocardial territory affected, Killip class on presentation [10], procedural variables (thrombolysis in myocardial infarction [TIMI] flow before and after the procedure [11], use of thrombectomy device, method of revascularization [PTCA or stenting], type of stents, number of stents, myocardial blush grade [MBG] [12] after revascularization and presence of other major non-culprit artery stenosis) and postprocedural variables (peak cardiac enzymes, echocardiography variables and short-term complications related to MI or PCI procedure).

CMR was done 2–4 days after PPCI at the Radiodiagnosis Department, Ain Shams University using a 1.5 Tesla superconductive MR scanner (Philips Achieva-XR Medical Systems, Best, the Netherlands) with dedicated phased array Cardiac coil for enhanced resolution and increased signal-to-noise ratio.

The basic protocol included were single-shot black blood (axial, sagittal and coronal views), Cine short axial views, Cine 4 chamber views, Dynamic fast field echo (FFE) and Lock Locker.

The main sequences that were interpreted in this study were:

- T2WI STIR (TR 2 beats, TE 80) (to detect the edematous changes in the form of bright signal intensity changes)
- Post-contrast imaging: gadolinium was administered as bolus of 0.1 mmol/kg then delayed imaging from 10 to 30 min according to lock locker. (To detect the ischemic territorial affection, infarct size, percentage of microvascular occlusion).

In patients with microvascular obstruction (MO), the dark areas were included within infarct size analysis and the area of MO was evaluated separately.

Then the following parameters were calculated:

- Area at risk = edema volume/volume of left ventricle (LV) mass.
- Percentage of infarct size (infarction fraction) = infarction volume/volume LV mass.
- Percentage of MO = volume MO/volume LV mass.
- Myocardial salvage = area at risk - infarct size.
- Myocardial salvage index (MSI) = area at risk - infarct size/area at risk.

The primary endpoint of the study was the occurrence of any major adverse cardiovascular events (MACE) defined as death or re-infarction or new-onset heart failure at 6 months follow-up after the index event.

### Statistical analysis

Data were analyzed using MedCalc© version 15 (MedCalc© Software bvba, Ostend, Belgium) and version 23 (IBM© SPSS© Statistics IBM© Corp., Armonk, NY, USA) and normality of numerical data distribution was examined using the D’Agostino-Pearson test. Non-normally distributed numerical variables were presented as median and interquartile range and between-group differences were compared by the Mann-Whitney test. Categorical variables were presented as the percentage and number. *P* value was used to determine the statistical significance. A value of 0.05 is arbitrary a cut-off value; below it, the relationship between variables is considered statistically significant, and above it, the relation is considered statistically not significant.

## Results

### Demographic and clinical characteristics

The studied population included 20 (80%) males. The median age group was 48.3 ± 8.6 years. Seventeen (68%) were smokers, 4 (16%) had a history of Tramadol drug addiction, 2 (8%) had a history of cannabis smoking. Eight (32%) were diabetics, 4 (16%) were hypertensive, 2 (8%) had dyslipidemia and 3(12%) had a family history of premature CAD. Regarding Killip class on presentation, 23 (92%) cases were classified as Killip class I, 2 (8%) were classified as Killip class II while none of our study population was presented with Killip class III or IV. ECG done on presentation showed 3.8 ± 1.8 mm ST-segment elevation with 1.8 ± 1.0 mm reciprocal ST-segment depression. Demographic and clinical data together with time intervals of clinical relevance are shown in [Table 1].

**Table 1:** Demographic and clinical data of our study population

Age (years)	48.3 ± 8.6 (30–65)
Male gender	20 (80%)
Smoking	17 (68%)
Tramadol addiction	4 (16%)
Cannabis addiction	2 (8%)
DM	8 (32%)
Hypertension	4 (16.0%)
Dyslipidemia	2 (8.0%)
Obesity	2 (8.0%)
Old DVT	1 (4%)
Family history of premature CAD	3 (12%)
Killip I	23 (92.0%)
Killip II	2 (8%)
ST elevation (mm)	3.8 ± 1.8
Reciprocal ST depression (mm)	1.8 ± 1
Pain-to-door time (hours)	5.6 ± 2.7
Door-to-balloon time (min.)	52.8 ± 67.2
Pain-to-balloon time (hours)	6.1 ± 3.6
Total pain time (hours)	6.9 ± 3.5
Time to peak cardiac enzyme level (hours)	13.7 ± 5.0
Pain-to-ST–segment resolution (hours)	7.8 ± 4.7

### Primary PCI procedural variables

Analysis of procedural variables showed that LAD was the culprit artery in 20 (80%) cases, RCA in 3 (12%) of cases and LCX in 2 (8%) of cases. Twenty-two (88%) patients had TIMI 0 flow in the culprit artery and 3 (12%) patients had TIMI 1 flow. Restoration of flow was done using PTCA in 15 (60%) patients and thrombus aspiration devices in 7 (28%) patients. Only one (4%) patient did not receive a stent, 21 (84%) patients received one stent and 3 (12%) patients received 2 stents. Only 2 DES were implanted and all other stents were BMS. All procedures ended up with regaining TIMI 3 flow. Fourteen (56%) patients had postprocedural MBG III in the affected territory, while 11 (44%) patients had MBG II. All PPCI procedural data are shown in [Table 2].

**Table 2:** Primary PCI variables

Restoration of flow	
No PTCA	9 (36%)
PTCA	15 (60%)
Wire only	1 (4%)
Thrombus aspiration	7 (28%)
Number of stents	
Nil	1 (4%)
One	21 (84%)
Two	3 (2%)
Stent type	
BMS	22 (88%)
DES	2 (8%)
TIMI flow before PCI	
TIMI 0	22 (88%)
TIMI I	3 (12%)
TIMI flow after PCI	
TIMI III	25 (100%)
MBG post-PCI	
Grade II	11 (44%)
Grade III	14 (56%)
Thrombus grading	
Grade III	4 (16%)
Grade IV	1 (4%)
Grade V	20 (80%)
Culprit vessel	
LAD	20 (80%)
LCX	2 (8%)
RCA	3 (12%)
Non-culprit vessel	
LAD	2 (8%)
LCX	4 (16%)
RCA	5 (20%)
D1	1 (4%)

### Postprocedural variables

**Fig. 1:**
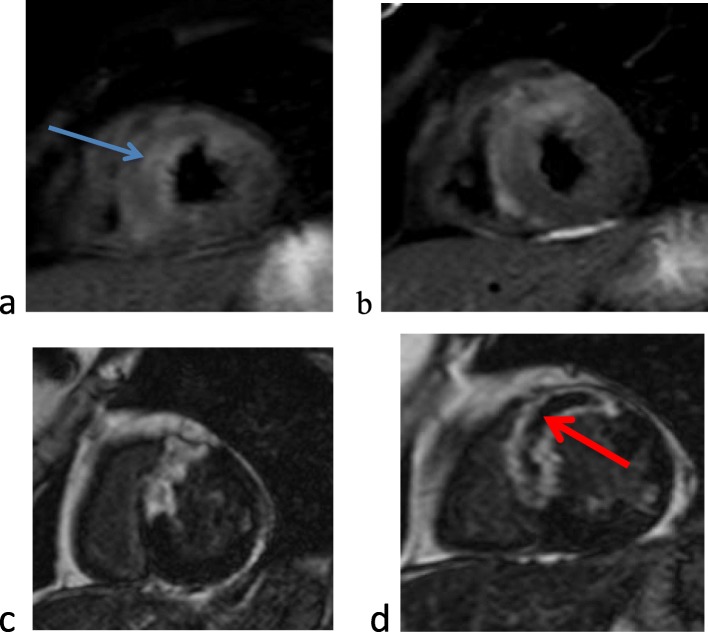
Case number 7 presented with anterior STEMI: **a**, **b** short-axis T2STIR images reveals edematous changes {blue arrow} involving LAD territory **c**, **d** short-axis delayed post-contrast images reveals transmural infarction involving LAD territory with large central MO {red arrow}

**Fig. 2:**
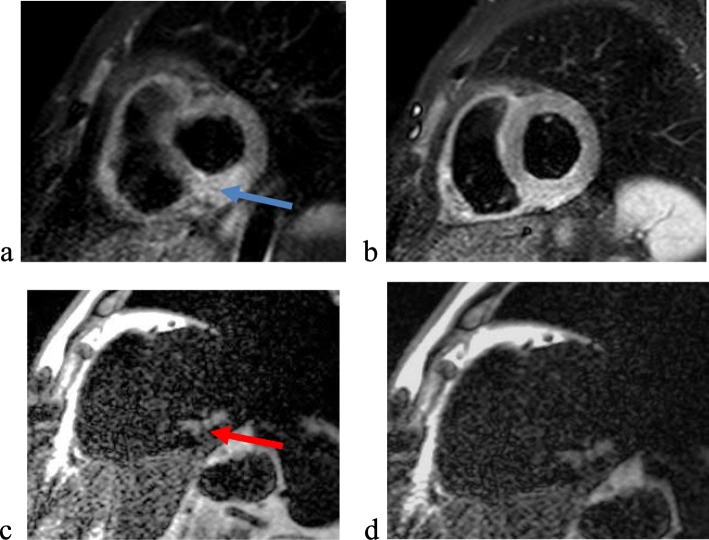
Case number 8 presented with inferior STEMI: **a**, **b** short-axis T2STIR images reveals edematous changes involving RCA territory {blue arrow}, edema volume 58,538 mm^3^ **c**, **d** short-axis delayed post-contrast images reveals transmural infarction involving RCA territory with no MO {red arrow}

In-hospital echocardiographic and CMR variables are shown in [Table 3]. Examples from our cases are shown in [Figs. 1 and 2]. Short-term MACE was encountered in the form of early stent thrombosis in 2 patients (8%) and anticoagulant-related hemorrhage which occurred in only one patient (4%). Long-term 6 months follow-up for the occurrence of MACE showed that 7 (28%) patients were affected. One patient suffered from re-infarction with acute heart failure, 3 patients suffered from re-infarction and 3 patients suffered from manifestations of heart failure.

**Table 3:** In-hospital echocardiographic and CMR variables

Echocardiography	
Normal diastolic functions	6 (24%)
DD Grade I	10 (40%)
DD Grade II	9 (36%)
LV thrombus formation	2 (8%)
EF on day 2 (%)	46 ± 9
CMR	
Volume of myocardial edema (mm^3^)	68,063.0 ± 34,057.0
Area at risk (fraction of LV mass)	0.419 ± 0.21
Infarction volume (mm^3^)	27,455 ± 22,094
Infarcted fraction (fraction of area at risk)	0.163 ± 0.112
MO volume (mm^3^)	4058.0 ± 6271.0
MO fraction (fraction of LV mass)	0.022 ± 0.033
Myocardial salvage fraction (fraction of area at risk)	0.255 ± 0.197
MSI	0.582 ± 0.275
LV cross-sectional area by echocardiography (mm^2^)	1916.6 ± 359.7
LV cross-sectional area by MRI (mm^2^)	4451.5 ± 2875.0
EF on day 2 by MRI (%)	52.0 ± 11

### Correlation between clinical, demographic, periprocedural characteristics and incidence of MACE

Apart from DM which showed a significant correlation with the incidence of MACE (*P* = 0.02), all other demographic, clinical and periprocedural variables did not show any significant relationship with this incidence.

### Correlation between CMR-derived parameters and incidence of MACE

Univariate analysis showed a significant correlation between edema volume, area at risk, infarction percentage and occurrence of MACE with *P* values of 0.04, 0.05 and 0.05 respectively. On the contrary, MSI failed to prove a significant correlation with MACE. Stepwise multivariate backward logistic regression analysis showed that infarction percentage is the most important CMR-derived parameter that can predict the occurrence of MACE (*P* = 0.05).

## Discussion

Management of patients with acute STEMI had enormously improved in the last decade and primary PCI became the standard of care for those patients [6, 7]. Despite this improvement in standards of care, mortality is still more than 7% during the first month after STEMI but progressively improves, although still considerable afterwards [5]. Cost-effective methods for early risk stratification are extremely needed for patient management by detection of high-risk patients and thereby incorporating those patients in short- and long-term follow-up programs to improve morbidity and mortality. CMR is one of these methods that is extremely appealing but unfortunately underused in Egypt mainly due to limited resources.

In this small study, we tried to start highlighting the role of different CMR-derived prognosticators in the management of this cohort of patients in Egypt. Our study was an observational prospective pilot study that included 25 patients who suffered an acute STEMI and were treated by PPCI. After 6 months of follow-up, 7 (28%) patients developed MACE.

We found that infarction percentage is the most important CMR-derived predictor for the occurrence of MACE. The amount of myocardium that is finally jeopardized by the acute ischemic insult has a direct impact on LV systolic functions which is up till now, the most important predictor of poor outcomes in this category of patients according to the most recent international guidelines [6, 7].

Our results in this context are agreeing with the results of the study done by Stiermaier et al. who used 2 separate patients’ cohorts to derive and validate a CMR-derived risk score to predict MACE in patients with acute STEMI treated by PPCI at 12 months interval. Infarction percentage ≥ 19% of LV size was one of the 3 components of this score [13].

MSI failed to prove a significant correlation with MACE in our study population. This was in contrary to the results obtained by Eitel et al., who showed that MSI is a strong predictor of MACE in these patients [14]. This difference might be attributed to the higher incidence of anterior STEMI in our study (80%) versus (47%) in their study. Patients with anterior STEMI can develop more complications even with a higher MSI due to the presence of a large affected territory. Also, the small number of our study population might have contributed to this discordance.

MO volume and percentage failed to prove any significant correlation with MACE in our study population. Our results in this context were different from those obtained by Symons et al. in their study which showed that MO detected by CMR at a median of 4 days after the index event is a strong predictor of poor outcomes in STEMI patients revascularized by PPCI, they also concluded that MO extent ≥ 2.6% of LV mass is a strong independent predictor of MACE [9]. Also, MO ≥ 1.4% of LV was one of the 3 components of the risk score developed and validated by Stiermaier et al. in their study [13]. In their study, Eitel et al. showed a similar significant predictive value of MO ≥ 1.4% of LV [15]. Again, Van Kranenburg et al. showed in their meta-analysis that included > 1000 patients who suffered STEMI in 8 CMR studies that MO can predict MACE independently [16].

Many studies were performed to understand the correlation between ischemic preconditioning, MO and its long-term effect after the acute event [17–19]. Many patients in developing countries experience prolonged ischemic preconditioning prior to developing an acute coronary syndrome more than their counterparts in developed countries due to lack of medical awareness and this might have decreased the significance of MO in our study versus other studies done in European and American centers. Also, there is no standard algorithm for LGE and MO quantification in STEMI and different protocols are used in different studies. Again, the small number of our study population might have affected our results.

Our study limitations were that our sample size was small; this made us unable to exclude the effect of other confounding factors on the occurrence of MACE. Our study was a single-center study and all our patients were Caucasian. Patients with cardiogenic shock and hemodynamic instability were excluded from our study. Longer follow-up intervals might be needed in future studies. Future studies are also needed in which PPCI is performed using DES only and preferably eluting the same drug from the same stent platform.

## Conclusions

Our study showed that infarction percentage is potentially the most important CMR-derived prognosticator in Egyptian patients with acute STEMI successfully reperfused by PPCI. It is also evident that despite having many studies in the past few years trying to figure out important CMR-derived parameters that affect prognosis in this category of patients, none of these studies gave solid data about which parameter is most important in prediction of outcome and there is no consensus till now about the most efficient prognosticator. Consequently; up till now, nothing of these parameters was incorporated in risk stratification models issued in the most recent guidelines of management for patients with STEMI [6, 7]. Rapid improvements in the technology, decreasing cost and standardization of study protocols will certainly pave the road for CMR to be included in future guidelines.

## Data Availability

The datasets used and analyzed during the current study are available from the corresponding author on reasonable request.

## Abbreviations

CAD: Coronary artery diseases
CMR: Cardiac magnetic resonance
EF: Ejection fraction
IHD: Ischemic heart disease
LGE: Late gadolinium enhancement
LV: Left ventricle
MACE: Major adverse cardiovascular events
MBG: Myocardial blush grade
MI: Myocardial infarction
MO: Microvascular obstruction
MSI: Myocardial salvage index
PPCI: Primary percutaneous coronary intervention
STEMI: ST-segment elevation myocardial infarction
TIMI: Thrombolysis in myocardial infarction
WHO: World Health Organization
